# A novel *PTH1R* mutation causes primary failure of eruption *via* the cAMP-PI3K/AKT pathway

**DOI:** 10.1186/s40510-025-00555-5

**Published:** 2025-02-24

**Authors:** Kejie Lu, Ying Qian, Jiaxing Gong, Zhiyong Li, Mengfei Yu, Huiming Wang

**Affiliations:** 1 Stomatology Hospital, School of Stomatology, Zhejiang University School of Medicine, Hangzhou, China; 2 Zhejiang Provincial Clinical Research Center of Oral Diseases, Key Laboratory of Oral Biomedical Research of Zhejiang Province, Hangzhou, China

**Keywords:** Primary failure of eruption (PFE), Parathyroid hormone 1 receptor (PTH1R), Affinity, Osteogenesis, Cell signaling

## Abstract

**Background:**

Primary failure of eruption (PFE) is a rare disorder characterized by a posterior open bite. While mutations in the parathyroid hormone 1 receptor (*PTH1R*) gene have been demonstrated to cause PFE, the underlying mechanisms remain largely unknown.

**Methods:**

Whole exome sequencing was conducted to identify *PTH1R* variants in a PFE family. MG63 cells that stably expressed the corresponding mutant PTH1R were established using lentiviruses. Next, osteogenesis was assessed by measuring cell alkaline phosphatase activity, conducting alizarin red staining, and evaluating osteoblast-specific gene expression. Then, computational analysis of binding affinity and RNA sequencing were carried out. Lastly, rescue experiments were performed to validate the mechanism underlying the pathogenesis of PFE.

**Results:**

A novel *PTH1R* missense mutation (c.904G > A, p.E302K) was identified in a Chinese family affected by PFE. Moreover, the E302K mutation inhibited the expression of osteogenic-specific genes and proteins in MG63 cells. Computational analysis revealed the E302K mutation decreased the binding affinity of Gα_s_ to the PTH1R protein. Consistently, cAMP accumulation assays demonstrated that the E302K mutation impaired the intracellular PTH_1-34_ -induced accumulation of cAMP. Further RNA sequencing analysis and validation experiments revealed that the PI3K-AKT signaling pathway was predominantly down-regulated in response to the E302K mutation. Finally, forskolin partially restored the effects of the E302K mutation on osteogenesis.

**Conclusions:**

This study indicated that the E302K mutation in PTH1R decreased the binding affinity of PTH1R protein for Gα_s_, down-regulated the cAMP-PI3K/AKT signaling pathway, and inhibited osteogenesis, eventually leading to PFE. This study not only expands the genotypic spectrum of *PTH1R* mutations but also elucidates the underlying pathogenic mechanism of *PTH1R*-associated PFE.

**Supplementary Information:**

The online version contains supplementary material available at 10.1186/s40510-025-00555-5.

## Introduction

As is well documented, primary failure of eruption (PFE [1] - OMIM#125350) is a rare disorder characterized by an abnormal eruption mechanism [2]. Its primary clinical manifestation is a posterior open bite in any number of quadrants. Moreover, PFE-affected teeth typically fail to respond to orthodontic traction, eventually leading to ankylosis [3]. PFE may be a genetic condition [4] associated with mutations in the parathyroid hormone 1 receptor (*PTH1R*) gene [5–8]. To date, over 50 different *PTH1R* variants have been identified in PFE patients [9].

The *PTH1R* gene is located on chromosome 3p21.31 (OMIM#168468), with a length of 26,054 bp and containing 16 exons. This gene encodes a 7-helical-transmembrane G-protein-coupled receptor, which can be bound to parathyroid hormone (PTH) and parathyroid hormone-related peptide (PTHrP) [10, 11]. Recent studies have identified a link between *PTH1R* gene alterations and functional impairment of tooth eruption [12, 13]. Takahashi et al. demonstrated that PTH1R deletion in PTHrP^+^ dental follicle cells resulted in molar eruption failure [14]. Interestingly, the aggravated open bite observed in adult mutant mice accurately simulated the primary features of human PFE [15]. Meanwhile, PTHrP_1-34_ injection accelerated tooth eruption [16]. Besides, earlier studies have identified several pathways downstream of PTHrP/PTH1R signaling, potentially involved in tooth eruption, including Wnt/β-catenin, Hh and TGF-β/BMP [17]. For example, TGF-β/BMP signaling in Osx^+^ mesenchymal cells indirectly regulates tooth eruption by modulating osteoblast differentiation and osteoclast formation [18, 19]. These findings collectively indicate that PTHrP/PTH1R signaling plays an essential role in tooth eruption. However, the mechanisms by which *PTH1R* mutations influence PTHrP/PTH1R signaling and key downstream pathways involved in PFE pathogenesis remain elusive.

In this study, a novel missense mutation (c.904G > A, p.E302K) in the *PTH1R* gene was identified in a Chinese family affected by PFE. Subsequently, PTH1R E302K mutation cell lines were constructed to assess osteogenesis. Moreover, the effects of the E302K mutation on the binding of Gα_s_ to the PTH1R protein were explored, while critical downstream signaling pathways that participate in the pathogenesis of PFE were identified and validated.

## Materials and methods

### Subject recruitment and mutational analyses

This study was approved by the Ethics Committee of Stomatology Hospital, School of Medicine, Zhejiang University, China, (No.2022 − 180(R)), with written consent obtained from all four participants. The girl (proband) seeking treatment at Affiliated Stomatology Hospital of Zhejiang University was initially diagnosed with PFE, characterized by eruption disturbances involving seven permanent teeth and the retention of one deciduous tooth, excluding the third molars. An experienced dentist examined the proband’s available lineal relatives. Other family members exhibited normal tooth morphology and count. Peripheral blood samples were collected from the proband, her sister, and her mother, while a saliva sample was collected from her father.

Next, genomic DNA was extracted from peripheral blood and saliva samples. Whole exome sequencing (WES) for the proband family was conducted by Annoroad Gene Biotechnology Co. Ltd (Zhejiang, China). Sanger sequencing was performed to verify the concordance of related fragments with WES results. The detailed procedures for DNA extraction, WES, and Sanger sequencing are described in the Supplementary Material 1.

### Computational analysis of PTH1R and PTH1R-Gαs complex

The three-dimensional structure of PTH1R in complex with PTH and Gs (PDB ID: 8FLQ) was retrieved from RCSB (https://www.rcsb.org). The docking process and molecular dynamics (MD) simulation were utilized to evaluate the impact of the E302K mutation on the binding of Gα_s_ to the PTH1R protein. Docking complexes were generated using Haddock 2.4, ClusPro 2.0, HDOCK, and pyDOCK. MD simulation was performed using GROMACS (v2022.2). All systems underwent a 1200-ns simulation during the MD production step. Structural parameters, including root-mean-square deviation (RMSD), root-mean-square fluctuation (RMSF), solvent accessible surface area (SASA), radius of gyration (Rg), minimum distance, and the number of contacts were derived from the output trajectory files. Procedures for structure preparation, docking analysis, and MD simulation are summarized in the Supplementary Material 1.

### Functional investigations of the E302K mutation

To establish MG63 cells stably expressing wild-type PTH1R or mutant PTH1R, lentiviruses (multiplicity of transfection = 100) were utilized for transfection. MG63 cells transfected with wild-type PTH1R, mutant PTH1R recombinant lentivirus, or GFP control lentivirus were designated as Wild-type, E302K, and GFP, respectively.

The experimental procedures for lentiviral transfection, cell culture, alkaline phosphatase (ALP) staining, quantification of ALP activity, alizarin red staining, real-time (RT)-qPCR, western blotting (WB), and intracellular cAMP measurement are detailed in the Supplementary Material 1.

### Downstream mechanistic investigations of the E302K mutation

Total RNA was isolated from GFP, Wild-type and E302K cells stimulated with PTH_1-34_ (GLPBIO). RNA-seq libraries were prepared using the NEBNext^®^ Ultra™ RNA Library Prep Kit (NEB, USA) and sequenced on Illumina at Novogene Co. Ltd. (Beijing, China). Gene ontology (GO) enrichment analysis was conducted for functional annotation. Pathway enrichment was analyzed using the kyoto encyclopedia of genes and genomes (KEGG) database. Gene set enrichment analysis (GSEA) was employed to examine gene pathways and datasets. The detailed procedures of transcriptome sequencing (RNA-seq) and data analysis are presented in the Supplementary Material 1.

## Results

### Features of clinical and radiographic reflected PFE

**Fig. 1:**
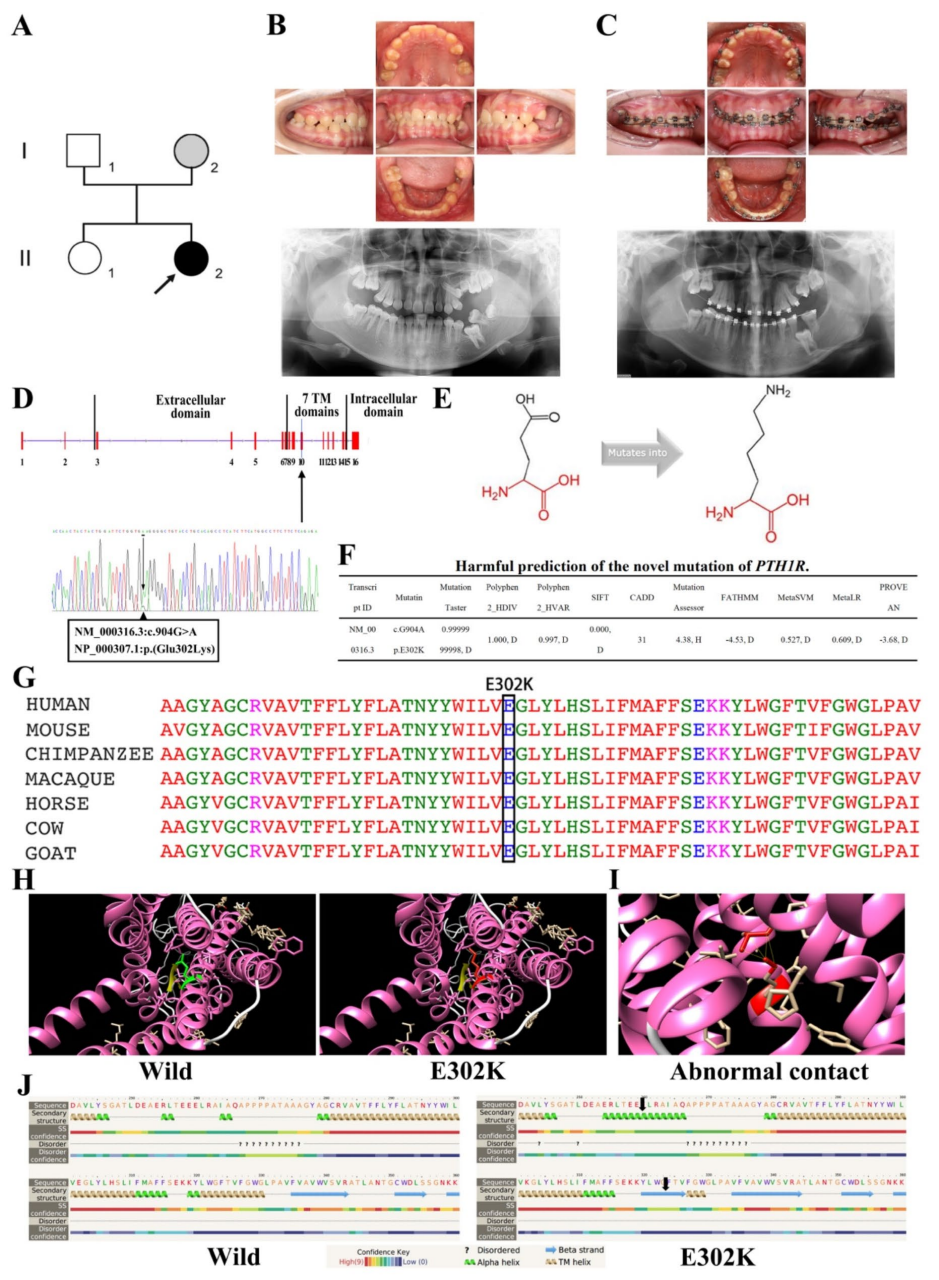
The family with a heterozygous missense mutation (*PTH1R*, c.G904A, p.E302K) causing PFE. (**A**) The family tree with affected proband (II:2) is indicated in black. Open symbols indicated unaffected individuals who did not carry the c.G904A variant. Individual I:2 (proband’s mother) was suggested in gray since she carried the same variant but did not express the phenotype. (**B-C**) Intraoral photographs and panoramic radiographs of the proband (II:2) at her first consultation and after the two-year orthodontic treatment. (**D**) Localization on exon 10 of the missense *PTH1R* variant identified in the proband (II:2). The sequence variant with the heterozygous variant G > A was shown as a double peak in the electropherogram. (**E**) The 904G > A mutation changed the amino acid at position 302 from glutamic acid to lysine. (**F**) The harmful prediction of the heterozygous missense mutation of *PTH1R*. (**G**) A conservation analysis of the mutation site. (**H-I**) Three-dimensional structure analysis of the wild-type and the mutant PTH1R. (**J**) Secondary structure analysis of the mutated PTH1R. Transformations are marked with black arrows

The proband (II:2/age 14) was the sole individual in the family (Fig. 1A), manifesting a characteristic PFE phenotype, that is, eruption failure of permanent teeth in the absence of mechanical obstructions and affected teeth not responding to orthodontic force [20]. Clinically, bilateral posterior open bites were observed in the proband (II:2). The panoramic radiograph displayed infra-occluded maxillary first and second molars in the absence of mechanical obstruction, whilst the mandibular first molar seemed to be obstructed by the second molar (Fig. 1B). Moreover, a two-year orthodontic treatment was unsuccessful in repositioning the affected teeth (17, 26) (Fig. 1C). On the other hand, clinical examination of other family members revealed no clinical signs of PFE. Nevertheless, it is worthwhile emphasizing that the proband’s mother (I:2) exhibited short stature.

### A novel pathogenic heterozygous missense mutation might impair the function of the PTH1R protein

Whole exome sequencing of all available family members identified a novel heterozygous missense variant (c.G904A) in the *PTH1R* gene at Chr3:46940862, present in both the proband (II:2) and her mother (I:2) (Fig. 1D). This pathogenic variant induced a substitution of glutamic acid (E) with lysine (K) at position 302 (p.E302K) (Fig. 1E). Importantly, the missense variant was categorized as deleterious by several prediction software programs (Fig. 1F) (MutationTaster score = 0.9999999998, Polyphen2_HDIV score = 1.000, Polyphen2_HVAR score = 0.997, SIFT score = 0.000, MutationAssessor score = 4.38, CADD = 31, FATHMM = -4.53, MetaSVM = 0.572, MetaLR = 0.609, and PROVEAN = -3.68). Moreover, the dbNSFP database demonstrated that glutamic acid at position 302 was highly conserved across species, with GERP++_RS and GERP + + GT2 scores of 5.43. (Fig. 1G).

To investigate the effect of pathogenic variants on protein function, schematic illustrations of the wild-type and mutant proteins were constructed (Fig. 1H). Analysis of secondary and tertiary structures of the mutated protein unveiled abnormal residue-residue contact between K302 and L226, accompanied by an increasing number of α-helices and β-strand structures (Fig. 1I-J). We hypothesized that these structural abnormalities resulted from the larger size and opposite charge of mutant amino acids. Finally, the results of sequence conservation analysis suggested that the mutation occurred in a crucial domain and potentially dysregulated the primary function of the protein.

### Construction of MG63 cells stably overexpressing wild-type or E302K PTH1R

**Fig. 2:**
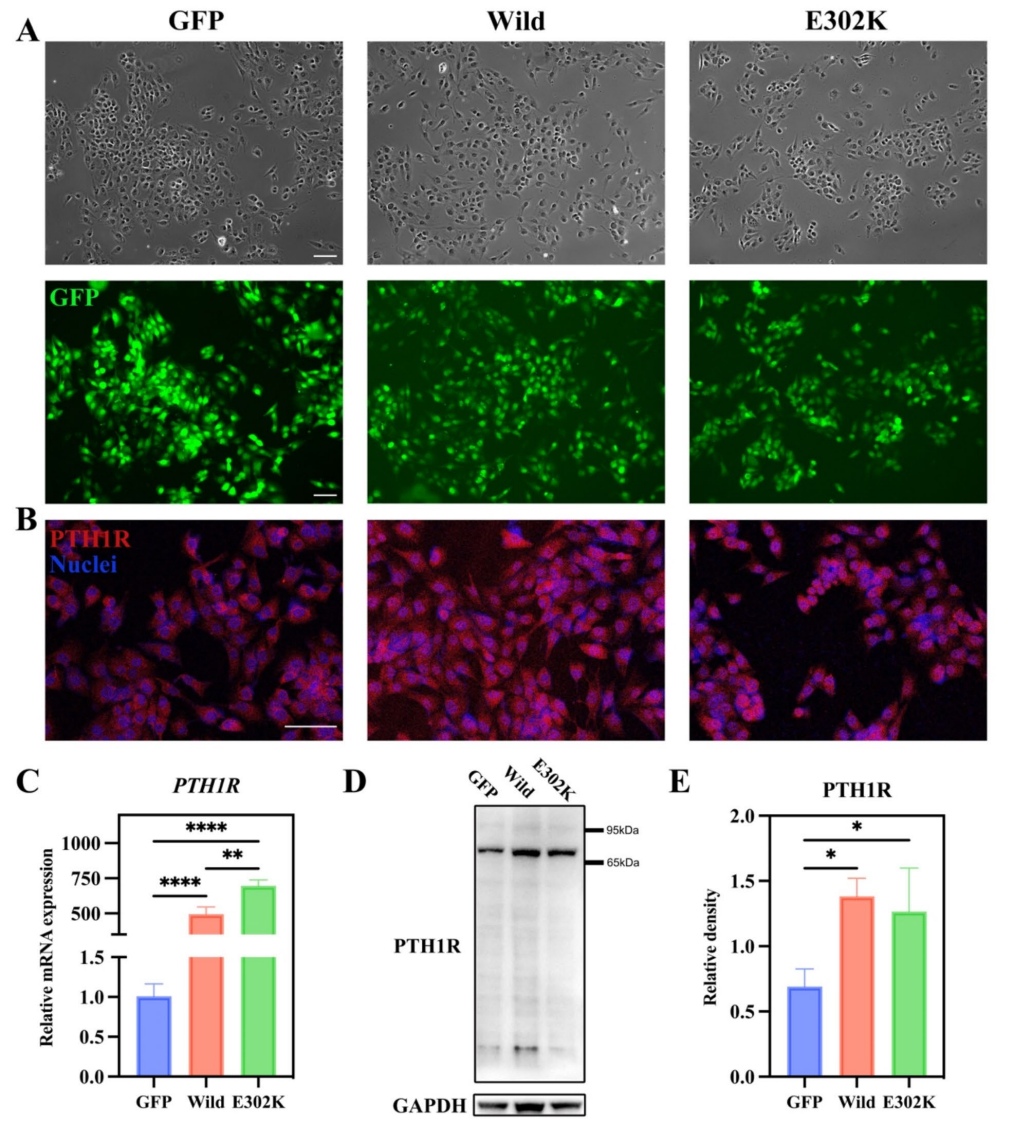
Overexpression of wild-type and mutant PTH1R in stably transfected MG63 cells. MG63 cells transfected with wild-type PTH1R, mutant PTH1R recombinant lentivirus or GFP control lentivirus were named Wild-type, E302K, and GFP, respectively. (**A**) GFP expression in stably transfected MG63 cells. The top row showed normal microscopic views of MG63 cells, and the bottom row indicated GFP expression, which was photographed by a fluorescence microscope. Scale bar = 100 μm. (**B**) Immunocytochemical analysis of PTH1R protein in MG63 cells transfected with wild-type and mutant PTH1R. Scale bar = 100 μm. (**C**) Quantitative analysis of the mRNA levels of *PTH1R* in stably transfected MG63 cells. (**D**) WB assays examined the protein level of PTH1R in MG63 cells. (**E**) The histograms showed the quantification of band intensities. *P < 0.05, **P < 0.01, ****P < 0.0001

Stable overexpression of wild-type or E302K PTH1R in MG63 cells was successfully established. Fluorescence analysis indicated that almost all stably transfected MG63 cells expressed GFP (Fig. 2A). As anticipated, the PTH1R fluorescence intensity was higher in the Wild-type or E302K groups compared to the GFP group (Fig. 2B). At the same time, the results of RT-qPCR showed that the mRNA expression level of *PTH1R* was higher by more than 400-fold in MG63 cells transfected with Wild-type or E302K groups compared to the GFP group (*P* < 0.0001) (Fig. 2C). Similarly, WB analysis demonstrated that the protein expression level of PTH1R was significantly higher in the Wild-type or E302K groups compared to the GFP group (Fig. 2D-E). These results conjointly indicated that wild-type PTH1R or mutant PTH1R was stably overexpressed in MG63 cells.

### E302K mutation inhibited osteogenesis in MG63 cells

**Fig. 3:**
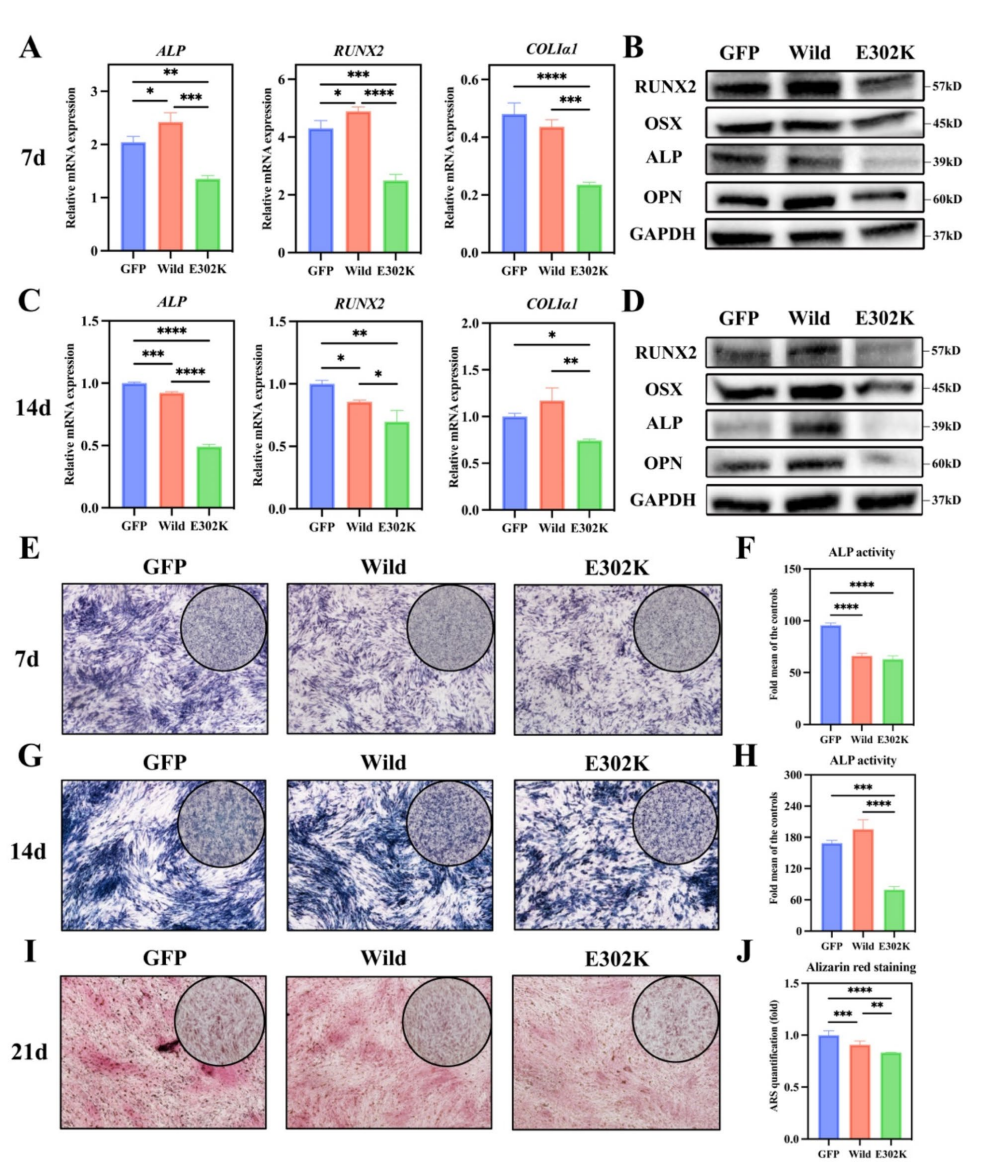
The E302K mutation inhibited the osteogenesis of MG63 cells. (**A, C**) Quantitative analysis of *ALP*, *RUNX2* and *COLIα1* mRNA levels in MG63 cells after the cells were cultured in osteogenic medium for 7 and 14 days. (**B, D**) WB assays analyzed RUNX2, OSX, ALP, and OPN protein levels in MG63 cells after the cells were cultured in an osteogenic medium for 7 and 14 days. (**E-H**) ALP staining and ALP activity of MG63 cells after induction in osteogenic medium for 7 days and 14 days. (**I, J**) Alizarin red staining of MG63 cells after induction in osteogenic medium for 21 days and quantification of alizarin red staining by spectrophotometry. Scale bar = 500 μm. *P < 0.05, **P < 0.01, ***P < 0.001, ****P < 0.0001

The impact of mutant PTH1R on osteogenesis was examined through ALP activity and staining, alizarin red staining, and the expression of osteogenic markers. After 7 and 14 days of osteogenic induction, RT-qPCR analysis revealed that the mRNA expression levels of osteoblast-specific genes (*ALP*, *RUNX2*, and *Col Iα1*) were lower in the E302K group compared to the Wild-type group (Fig. 3A, C), consistent with the results of WB analysis (Fig. 3B, D). ALP staining revealed that ALP expression levels were significantly lower in the E302K group compared to the Wild-type group following 14 days of osteogenic induction (Fig. 3G). In contrast, no significant difference was observed after 7 days of osteogenic induction (Fig. 3E). As expected, the results of ALP activity assays were consistent with those of ALP staining (Fig. 3F, H). Alizarin red staining analysis revealed a lower number of mineralized nodules in the E302K group compared to the Wild-type group following 21 days of osteogenic induction (Fig. 3I, J). Overall, these results suggested that the E302K mutation impaired osteogenesis in MG63 cells.

### The E302K mutation inhibited Gα_s_ signaling by weakening the binding affinity of Gα_s_ to the PTH1R protein

**Fig. 4:**
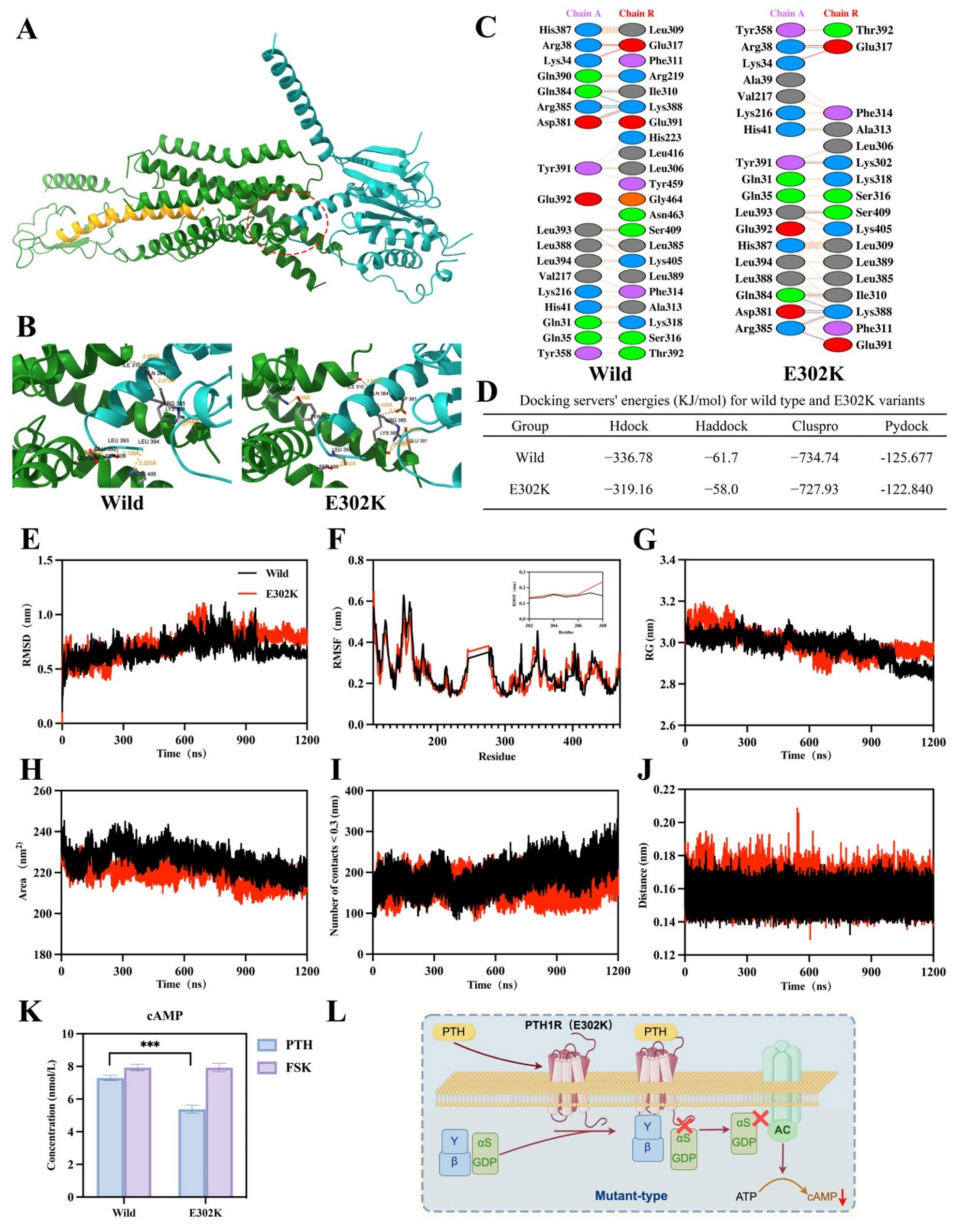
The E302K mutation weakened the binding affinity of Gα_s_ to the PTH1R protein. (**A**) Binding interface of PTH1R-Gα_s_ complexes. (**B**) 3D visualizations of H bond numbers and distances. (**C**) Representation of interface interactions using the PDBsum server. (**D**) Docking servers’ energies (KJ/mol) for wild-type and E302K variants. (**E-F**) RMSD and RMSF curves during 1200 ns MD simulations for PTH1R-Gα_s_ complexes. As can be seen, after 900 ns, the RMSD value reached a steady state, showing that the simulation time is sufficient for systems equilibration. The RMSF for amino acids at positions 302 to 308 is detailed in a gray square. (**G-H**) The Rg and the SASA plots over 1200 ns MD simulations of the E302K (red) and Wild-type (black). (**I-J**) Number of contacts and the minimum distance for interface residues of PTH1R-Gα_s_ complexes in both E302K (red) and Wild-type (black) forms. (**K**) Intracellular accumulation of cAMP after treatment with PTH_1-34_. Wild-type and E302K cells were treated for 15 min with 25 nmol/L PTH_1-34_ in the presence of 1 mmol/L IBMX. As a positive control of cAMP production, cells were treated for 15 min with 50 µmol/L forskolin in the presence of IBMX. ***P < 0.001. (**L**) Schematic diagram of the mechanism of the E302K mutation inhibited Gα_s_ signaling

MD was performed to elucidate the mechanism by which the E302K mutation impacts intracellular signaling. The binding pattern between Gα_s_ and the mutant PTH1R protein displayed fewer interface residues, smaller interface area, and longer hydrogen bond distance (Fig. 4A-C). The results of the four protein docking softwares indicated that the E302K variant exhibited stronger binding free energy, leading to a reduced binding affinity between PTH1R and Gα_s_ (Fig. 4D). To further analyze their interaction, MD simulation was carried out. The average RMSD of the Wild-type protein and the E302K protein was 0.670 nm and 0.814 nm, respectively (Fig. 4E), signifying that the E302K protein was less stable than the Wild-type protein. Additionally, the structural fluctuations around the 302nd residue of the E302K protein were marginally higher compared to the Wild-type protein (Fig. 4F). As depicted in Fig. 4G, the Rg profile averaged 2.896 nm for the Wild-type and 2.954 nm for the E302K variant, indicating decreased compactness and reduced interactions in the E302K protein. The average SASA values for the Wild-type and E302K were 219.98 and 214.20 nm, respectively (Fig. 4H), implying the substitution of glutamic acid to lysine at position 302, leading to a lower interaction power between PTH1R and Gα_s_. The Wild-type-Gα_s_ complex exhibited mean, minimum, and maximum contact numbers of 220.3, 137.0, and 332.0, respectively. In contrast, the E302K variant showed lower values of 160.2, 94.0, and 263.0, respectively (Fig. 4I). Meanwhile, the E302K variant showed a greater distance of 0.158 nm compared to the Wild-type’s 0.155 nm (Fig. 4J). The aforementioned findings indicated that E302K exhibited reduced binding affinity for Gα_s_ and weakened interactions within the E302K-Gα_s_ complex, potentially diminishing Gα_s_ signaling. Furthermore, Gα_s_ activation was examined by measuring PTH_ 1-34_-induced cAMP accumulation in the Wild-type and E302K groups. The cAMP levels in E302K cells were lower than in Wild-type (Fig. 4K). Thus, the E302K mutation contributed to the low binding affinity of PTH1R to Gα_s_, inhibiting adenylate cyclase (AC), reducing intracellular levels of cAMP and impeding downstream Gα_s_ signaling (Fig. 4L).

### The E302K mutation inhibited osteogenesis *via* the cAMP-PI3K/AKT signaling pathway

**Fig. 5:**
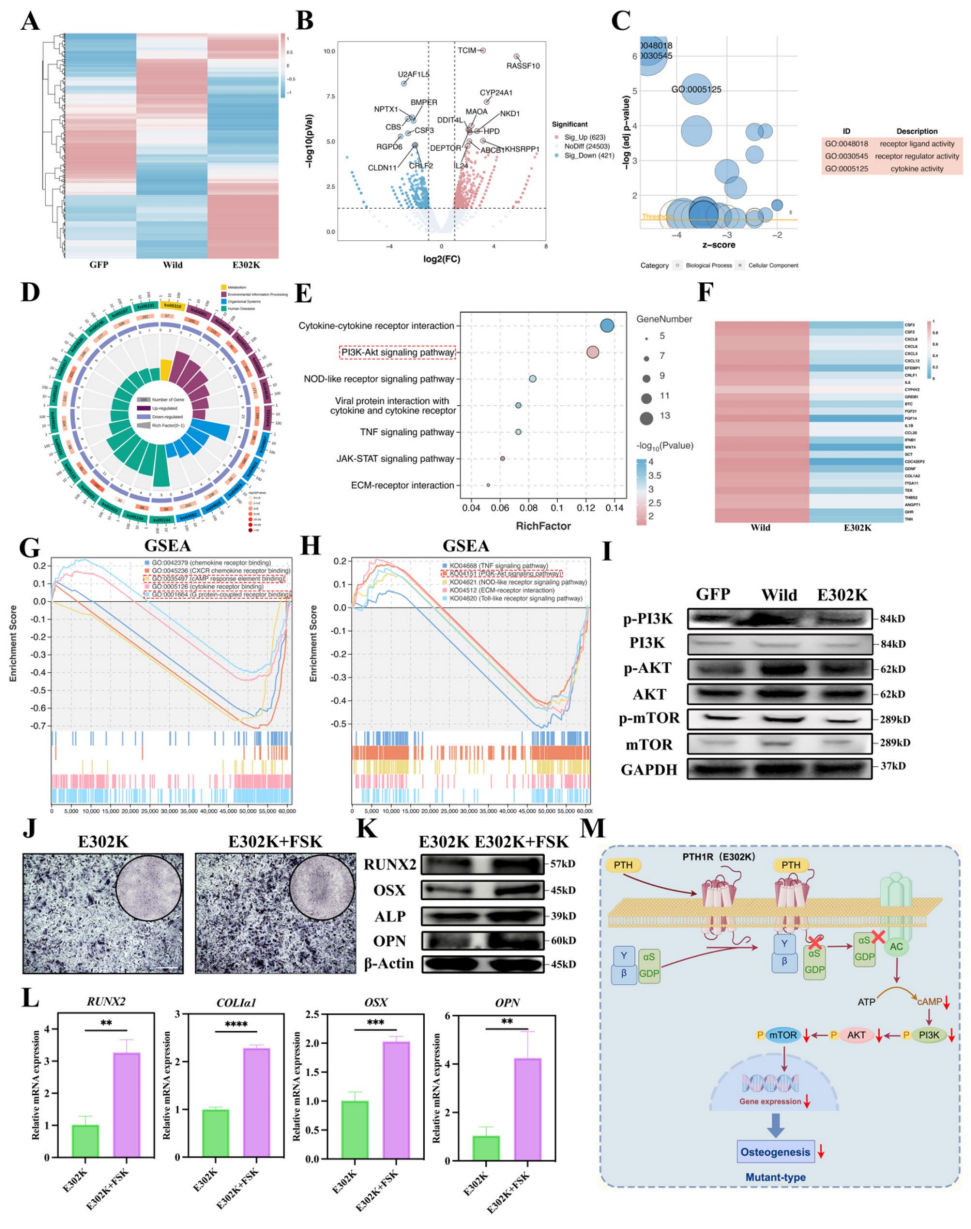
The mechanism of the E302K mutation inhibited osteogenesis. (**A**) RNA-seq analysis of GFP, Wild-type and E302K cells and gene expression profiles were presented in a heatmap. (**B**) Volcano plots of differentially expressed genes in Wild-type and E302K cells. (**C**) GO analysis of the most differentially expressed gene sets in Wild-type and E302K cells. (**D**) KEGG pathway analysis of enriched signaling pathways in Wild-type and E302K cells. (**E**) Signaling pathway analysis indicated a different enrichment of the PI3K-AKT signaling pathway. (**F**) Heatmap of differentially expressed genes related to PI3K-AKT signaling pathway in Wild-type and E302K cells. (**G**) GSEA of GO analysis in Wild-type and E302K cells. (**H**) GSEA of KEGG pathway analysis in Wild-type and E302K cells. (**I**) WB assays analyzed the protein level of p-PI3K, PI3K, p-AKT, AKT, p-mTOR and mTOR in GFP, Wild-type and E302K cells after the cells were cultured with PTH_1-34_. (**J**) ALP staining of E302K cells treated with FSK after induction in osteogenic medium for 7 days. (**K**) WB assays analyzed the protein levels of RUNX2, OSX, ALP, and OPN in E302K cells treated with FSK after the cells were cultured in an osteogenic medium for 7 days. (**L**) Quantitative analyses of *RUNX2*, *COLIα1*, *OSX* and *OPN* mRNA levels in E302K cells treated with FSK after the cells were cultured in osteogenic medium for 7 days. (**M**) Schematic diagram of the mechanism of the E302K mutation inhibited osteogenesis. Scale bar = 500 μm. **P < 0.01, ***P < 0.001, ****P < 0.0001

To identify key signaling pathway(s) downstream of Gα_s_ responsible for the occurrence of PFE, RNA-seq was performed on GFP, Wild-type and E302K cells stimulated with PTH_1-34_. Differentially expressed genes are presented in the hierarchical clustering heat map and volcano plot (Fig. 5A-B). Subsequently, the GO online tool was utilized to identify pathways enriched in the Wild-type and E302K groups. The downregulated pathways, encompassing receptor-ligand activity, receptor regulator activity, and cytokine activity, were significantly enriched in the E302K (Fig. 5C), in agreement with the results obtained from the MD simulation. Furthermore, KEGG database analysis revealed the association of downregulated genes with human diseases, environment information processing, and organismal systems (Fig. 5D). Among them, the PI3K-AKT signaling pathway, associated with Gα_s_ signaling, exhibited the highest gene enrichment (Fig. 5E). Of note, 28 differentially expressed genes were identified between the Wild-type and E302K groups. Specifically, these genes exhibited relatively low expression levels in the E302K (Fig. 5F). Next, GSEA was performed to explore GO pathways. The results signaled the E302K mutation regulated cAMP response element binding and G protein-coupled receptor binding, which was consistent and complementary with the GO analysis (Fig. 5G). Furthermore, GSEA of KEGG pathways revealed enrichment of the PI3K-AKT signaling pathway in the E302K group (Fig. 5H). Thereafter, WB analysis was undertaken to evaluate the protein levels of PI3K, p-PI3K, AKT, p-AKT, mTOR, and p-mTOR, validating the sequencing results. Our findings demonstrated that the E302K mutation down-regulated the expression of p-PI3K, p-AKT, and p-mTOR (Fig. 5I). To further investigate the impact of the E302K mutation on osteogenesis and the cAMP-PI3K/AKT pathway, rescue experiments were performed using forskolin (FSK), an AC activator. As illustrated in Fig. 5J, K, and L, ALP staining, RT-qPCR and WB assays revealed that the E302K + FSK group exhibited significantly enhanced osteogenesis compared to the E302K group. Taken together, these findings indicate that the E302K mutation inhibited osteogenesis by modulating the cAMP-PI3K/AKT signaling pathway (Fig. 5M).

## Discussion

This study identified a novel missense mutation (c. 904G > A, p. E302K) in the *PTH1R* gene of a PFE family. Herein, the influence of the E302K mutation on the osteogenic capability and *PTH1R*-related signaling pathways was explored *via* stable overexpression of E302K in cells, MD simulation, and RNA-seq.

PFE is a rare disease hallmarked by localized tooth eruption failure without identifiable local or systemic causes [21]. Herein, the proband (II:2) was the sole family member exhibiting a characteristic PFE phenotype, marked by the failure of first molars and other posterior teeth to erupt, retained deciduous teeth, and no response to orthodontic force. Besides, the absence of mechanical obstruction in the eruption path of permanent teeth suggested an unidentified cause for the delayed tooth eruption. These findings corroborated the possibility of PFE and genetic involvement, warranting genetic testing. Genetic analysis identified a novel missense mutation (c. 904G > A, p. E302K) in the *PTH1R* gene within this family. Noteworthily, the proband (II:2) and her unaffected mother (I:2) harbored the same variant. Moreover, the proband’s mother (I:2) presented with short stature. This finding highlighted the known phenomenon of reduced penetrance, as observed in some PFE families [20, 22, 23]. Reduced penetrance, an extreme form of variable expressivity, likely results from the crosstalk between genetic, epigenetic, and microenvironmental factors, affecting the same pathogenic pathway uniquely for each individual [24]. Studies in Caenorhabditis elegans concluded that incomplete penetrance might result from ‘random fluctuations’ in gene expression [25]. The type of pathogenic mutation, whether missense, nonsense, or frameshift, does not markedly influence penetrance expression, with incomplete penetrance being caused by haploid insufficiency of the pathogenic gene [26]. Therefore, we speculated that the short stature of the proband’s mother (I:2) might be a result of PTH1R haploinsufficiency. A similar phenomenon has been described in previous studies [20]. The PTH1R protein, a 7-helical-transmembrane G-protein-coupled receptor, predominantly mediates bone metabolism in response to PTH and PTHrP [27]. It can be divided into three domains, namely the extracellular N-terminal domain, the J domain (composed of the transmembrane helices and a connecting loop), and the intracellular C-terminal domain [28]. This study noted that the E302K mutation, located in the highly conserved J domain, led to abnormal residue-residue contact and an increase in α-helices and β-strand structures. We posited that the E302K mutation might impair PTH1R protein function, such as bone remodeling.

During tooth eruption, bone formation and bone resorption are required chronologically and spatially [29]. The former occurs at the base of the alveolar bony crypt [30]. Bone formation at the base of the socket has been identified as a potential driving force that promotes tooth eruption [31]. The present study demonstrated that the mutant PTH1R disrupted ALP activity and hindered the formation of mineralized nodules. These results were somewhat predictable. Jr et al. evinced that conditional deletion of the *PTH1R* in osteocytes facilitated the development of osteopenia in mice [32] and significantly decreased the alveolar bone volume and bone growth rate in PTH1R-ablated mice [33]. More importantly, previous studies have established that PTH1R directly mediated signaling pathways to promote the osteoblastic differentiation of MSCs [34]. In another study, PTH1R over-expression enhanced osteoblast-related gene expression and promoted hDFCs osteogenesis [35], in line with the findings of this study. The E302K mutation significantly down-regulated the expression of osteogenic-specific genes (*ALP*, *RUNX2* and *Col Iα1*) and proteins (ALP, RUNX2, OSX, and OPN), implying that the E302K mutation might reverse the promoting effects of PTH1R on osteogenic differentiation.

How does the E302K mutation impair the osteogenic capability of cells? Previous studies have pointed out that mutations affect the location and structure of PTH1R, eventually culminating in PFE [36]. Hariharan et al. reported that the G452E PTH1R mutant was largely retained intracellularly, whereas wild-type PTH1R was expressed on the cell surface [37]. In addition, mutations in *PTH1R* are frequently associated with the absence, misfolding, or degradation of critical structures, leading to functionally inactive PTH1R [38]. For instance, 1092delG and 996_997InsC mutations particularly affect the regions of the third intracellular loop and the sixth transmembrane domain, which are required for efficient PTH1R function [39]. Earlier studies have reported that the PTH-induced activation of AC in osteoblasts plays a critical role in PTH1R signaling and influences bone formation [40]. Therefore, it is reasonable to hypothesize that different mutations in PTH1R might lead to variations in the affinity of PTH to PTH1R conformations. PTH has been documented to activate PTH1R *via* a ‘two-domain’ model [41]. PTH1R activation is initiated with the rapid binding of the C-terminal of PTH to the extracellular N-terminal domain, followed by the gradual insertion of the N-terminal of PTH into the J domain [42]. Furthermore, the impact of the E302K mutation on PTH1R’s PTH binding capability was examined. Regrettably, the mutant PTH1R possessed a PTH binding capacity similar to the wild-type protein (Supplementary Fig. 1). Thus, we further focused on Gα_s_, a stimulatory subunit of heterotrimeric G proteins that mediates PTH1R signaling by activating AC and generating cAMP [43]. Gα_s_ has been hypothesized to play a decisive role in bone development by regulating the differentiation [44] and mineralization [45] of osteoblasts. According to the results of the MD simulation, a lower interface area, fewer contacts, and an increase in mean distance in the E302K-Gα_s_ complex resulted in a weaker interaction, which lowered the binding affinity of PTH1R to Gα_s_. Moreover, Gα_s_ activation was examined by assessing intracellular cAMP levels in vitro experiments, and the results showed that cAMP levels were approximately 26% lower in the E302K group compared to the Wild-type group. Taken together, these results indicated the E302K mutation inhibited Gα_s_ signaling. Next, a transcriptome analysis of Wild-type and E302K cells stimulated with PTH_1-34_ was performed. The “receptor ligand activity”, especially “G protein-coupled receptor binding”, was one of the significantly enriched GO terms, consistent with the reduction in the binding affinity of Gα_s_ to E302K protein. Furthermore, KEGG and GSEA enrichment analyses corroborated that the PI3K-AKT signaling pathway was predominantly down-regulated in the E302K group, which was further validated at the protein level. At present, the PI3K-AKT signaling pathway has been found to play a pivotal role in osteogenic differentiation and bone growth [46, 47]. Indeed, activation of the PI3K-AKT signaling pathway promoted the osteoblastic differentiation of bone marrow MSCs or preosteoblasts into osteoblasts [48]. In the current study, the application of LY294002, a specific inhibitor of PI3K/AKT, suppressed bone formation [49]. FSK serves as an activator of AC, inducing the generation of intracellular cAMP and subsequent activation of the PI3K/AKT signaling pathway [50]. Herein, the observations indicated that FSK partially mitigated the suppressive effects of the E302K mutation on osteogenesis. Based on these results, we theorized that the E302K mutation impaired the PTH1R-Gα_s_ protein binding domain, disrupting the downstream cAMP-PI3K/AKT signaling pathway, which was associated with the development of PFE in our patient. Nevertheless, further in vivo models are necessitated to elucidate the specific molecular mechanism underlying PFE caused by the E302K mutation.

## Conclusion

In summary, the E302K mutation in PTH1R impaired the PTH1R-Gα_s_ protein binding domain, disrupting the downstream cAMP-PI3K/AKT signaling pathway and leading to osteogenic deficiency, which was associated with the development of PFE. These findings not only expand the genotypic spectrum of *PTH1R* mutations but also elucidate the underlying pathogenic mechanism of *PTH1R*-associated PFE.

## Electronic supplementary material

Below is the link to the electronic supplementary material.

Supplementary Material 1

Supplementary Material 2

## Data Availability

The datasets used and/or analyzed during the current study are available from the corresponding author on reasonable request.
