# Supplementary material for: A novel PTH1R mutation causes primary failure of eruption via the cAMP-PI3K/AKT pathway
Source: Prog Orthod. 2025 Feb 24;26:7. doi: 10.1186/s40510-025-00555-5 (PMC11847765; doi:10.1186/s40510-025-00555-5)
Supplement: Supplementary file 1 — Supplementary Material 1 [file 40510_2025_555_MOESM1_ESM.docx]

**Supplementary Material 1**

**A Novel *PTH1R* Mutation Causes Primary Failure of Eruption**

**via the cAMP-PI3K/AKT Pathway**

**Contents:**

**Supplementary Materials and Methods**

**Supplementary Figure 1.** The E302K mutation did not affect the binding affinity of PTH to the PTH1R protein.

**Supplementary Materials and Methods**

**DNA extraction and quantification**

Genomic DNA was isolated from peripheral blood and saliva samples using the QIAmp DNA Blood Midi Kit (Cat # 51185, Qiagen, Germany) and BioG Saliva DNA Isolation Kit (Cat #51043, Biogen, China), respectively, according to the manufacturer’s instructions. The concentration and purity of DNA samples were precisely quantified using a NanoPhotometer spectrophotometer (IMPLEN, CA, USA) and a Qubit 3.0 Flurometer (Life Technologies, CA, USA). DNA degradation and suspected RNA and protein contamination were verified by electrophoresis on 1% agarose gels.

**Whole-exome sequencing**

WES was performed by Annoroad Gene Biotechnology Co. Ltd (Zhejiang, China). Briefly, the exome sequences were efficiently enriched by applying a liquid capture system (Agilent SureSelect Human All Exon V6) by operational norms. Then, exons were captured using the SureSelectXT Target Enrichment System, real-time PCR amplification was conducted, and the library was subjected to quality inspection. Finally, a qualified DNA library was sequenced at the HiSeq sequencing platform. Filtered variants were prioritised using comprehensive analyses including mutation type (nonsynonymous variations in the coding region and splice site), the frequency of the variant (minor allele frequency ≤ 0.01 in the 1000 Genomes Project, the NHLBI GO ESP 6500 database, the ExACV3.1 database and the Kaviar_AF database), reported functions, and involved pathways of the relevant genes. Next, the deleteriousness of candidate mutations was predicted using REVEL [1], Mutation Taster [2], Polyphen2 [3], SIFT [4], Mutation Assessor [5], PROVEAN [6], MetaSVM, MetaLR, and CADD [7].

**Sanger sequencing**

Related fragments were tested for concordance by Sanger sequencing to further verify WES results. The nucleotide sequence of *PTH1R* was obtained from GenBank (Accession number: NC_000003.12; Gene ID: 5745). Primer sequences, designed using Primer3Plus online software, were as follows: 5′- TGCCCTCTGACTAACACCAG-3′(forward) and 5′- CTCTCTCCTGCTGTTCCACT -3′(reverse). PCR was performed using 1 µg of template DNA, 10 µM of each primer and 20 µL of the 2×T8 High-Fidelity Master Mix (TSE111, Tsingke, China) in a total reaction volume of 40 µL. The products were then sequenced using an ABI 3730xl Automatic Sequencer (Applied Biosystems, USA) by Tsingke Corporation (Wuhan, China).

**Computer analysis of PTH1R and PTH1R-Gα_s_ complex**

Preparation of structures

The three-dimensional structure of the PTH1R in complex with PTH and Gs (PDB ID: 8FLQ) was retrieved from the RCSB (https://www.rcsb.org). The protein preparation steps included removing water, ligands, and other complexed molecules, assigning proper bond orders, correcting disoriented groups, adding hydrogen atoms, and implementing relaxation and repack process for backbone and sidechains clashes fixation.

Docking and interaction analysis

The docking process was applied to consider the effect of the E302K mutation on the ability of Gα_s_ to PTH1R protein. Haddock 2.4 [8], ClusPro 2.0 [9], HDOCK [10] and pyDOCK [11] were used to attain the docking complexes. Through the docking, the solvated docking mode was activated, active residues were considered the fully flexible segment, and passive residues were chosen automatically. The cluster with the best conformation and highest score obtained in this step was employed in other analysis steps. The 3D interactions were created using the ChimeraX 1.6.1 [12] and SWISS-MODEL online software [13] (https://www.swissmodel.expasy.org) while the 2D interactions were created using LigPlot^+^ 2.2 [14] and PDBsum [15].

Molecular dynamics (MD) simulation

MD simulation was accomplished with the GROMACS (version 2022.2). Pressure, temperature, and neutralising conditions for the equilibrium of systems were checked before starting the MD analysis. Two trajectories were analysed for 1200 ns, 2 fs per step. As the simulation box, the boundary box to protein distance was considered a minimum of 1 nm. The desired structures were parameterised during the analysis with an OPLS-AA/L all-atom force field. The simulated protein complexes were placed in a cubic box, and the SPC/E water molecule was in the system. Na+ and Cl- ions were added to neutralise the net charge of systems. All the MD simulations were carried out considering periodic boundary conditions. The systems were energy minimised for 50000 steps using the steepest descent algorithm. The temperature was set at 300 K, and employed the velocity-rescaling thermostat during the simulation. Afterwards, the pressure was equilibrated at 1 bar using the Berendsen barostat. In the MD production step, all systems were simulated for 1200 ns. Structural parameters such as root-mean-square deviation (RMSD), root-mean-square fluctuation (RMSF), solvent accessible surface area (SASA), the radius of gyration (Rg), minimum distance, and the number of contacts were obtained from the output trajectory files.

**Cell culture**

Human osteosarcoma MG63 cells were obtained from the cell bank of the Guangzhou Biotechnology and cultivated in DMEM supplemented with 10% foetal bovine serum (FBS, Gibco, USA) and antibiotics (1% penicillin/streptomycin) at 37 °C in a humidified atmosphere with 5% CO_2_. For osteogenic induction, transfected MG63 cells were cultured in Oricell™ Kit (Cyagen Biosciences, USA), which included DMEM supplemented with 10% fetal bovine serum, l-ascorbic acid (0.5 mM), β-glycerophosphate (10 mM), and dexamethasone (0.01 mM). For PTH treatment, transfected MG63 cells were incubated in the presence of PTH_1-34_ (50 nmol/L) in the complete medium. For forskolin (FSK) treatment, transfected MG63 cells were incubated in the presence of FSK (3 µmol/L) in the osteogenic medium.

**Lentivirus construction and establishment of stably transfected** **MG63 cells**

For generation of wild-type PTH1R and mutant PTH1R (c.904G>A, p.E302K) overexpression lentiviruses, wild-type *PTH1R* and mutant *PTH1R* cDNA were separately subcloned into the PGMLV-CMV-MCS-3×Flag-EF1-ZsGreen1-T2A-Puro. The recombinant and packaging vectors were transfected into 293 T cells to produce the lentivirus. The supernatant was harvested, filtered, and concentrated 2 days after transfection. Lentivirus containing a green fluorescent protein (GFP) tag without the target gene was used as a negative control. To establish stably expressing wild-type PTH1R or mutant PTH1R in MG63 cells, lentiviruses (multiplicity of transfection = 100) were used to transfect MG63 cells. Then, the transfected cells were selected in 1 µg/ml puromycin (GlpBio) for 7 days. Stably transfected MG63 cells were observed under a fluorescence microscope to determine the ratio of GFP-positive cells, which reflects the transfection efficiency. Real-time PCR and Western blotting were used to detect the expression of PTH1R in stably transfected MG63 cells. MG63 cells transfected with wild-type PTH1R, mutant PTH1R recombinant lentivirus or GFP control lentivirus were defined as Wild, E302K, and GFP, respectively.

**ALP** **staining and quantification of** **ALP activity**

MG63 cells were seeded at a density of 4 **×** 10^4^ cells/well in 12-well plates. When 80% confluence was reached, the medium was changed to Oricell™ Kit and cultured for 7 and 14 days. At the indicated time, ALP staining was performed using the NBT/BCIP staining kit (Beyotime, China) according to the manufacturer’s instructions. Cells were washed 3 times with PBS, fixed with 4% paraformaldehyde (Biosharp, China) for 15 minutes, and a BCIP/NBT working solution was added. The staining solution was discarded after being incubated for 15 minutes at room temperature. Cells were observed under an inverted microscope (Zeiss, Germany). The alkaline phosphatase was stained blue.

According to the manufacturer's instructions, the ALP activity was analysed using Lab Assay ALP (FUJIFILM Wako, Japan). The absorbance at 562 nm was measured using a spectrophotometric instrument (TECAN, Switzerland). Protein concentration was determined by a BCA kit (Beyotime, China), which was used to normalise the ALP activity. The ALP activity was calculated relative to that of the control group.

**Alizarin red staining**

MG63 cells were placed in 12-well plates and induced for 21 days in OM. The formation of mineralised matrix nodules was determined by alizarin red staining. MG63 cells were rinsed 3 times with PBS, fixed in 4% paraformaldehyde for 1 h at 37°C, and washed with PBS 3 times. Then, the cells were incubated using 40 mM alizarin red S (Cyagen Biosciences) for 20 min at 37°C. After washing with PBS twice to rinse needless unbound stains, the stained matrix was photographed with an inverted microscope (Zeiss, Germany). For quantification of matrix mineralisation, the stained samples were eluted in 100 mM cetylpyridinium chloride (Aladdin) for 1 hour to solubilise the alizarin red into the solution. Then, the absorbance at 562 nm of the released alizarin red was measured by a spectrophotometric instrument (TECAN, Switzerland). The alizarin red intensity was calculated relative to that of the control group.

**Real time (RT)-qPCR**

RT-qPCR was used to detect the transcriptional levels of osteogenic and osteoclast differentiation. Total RNA was extracted and purified by TRIzol reagent (Invitrogen); after that, the concentration was determined using the Nanodrop 2000c instrument (Thermo Scientific, USA). The mRNA was then reverse-transcribed into cDNA and expanded 40 times according to the manufacturer's recommendation (TaKaRa, Japan). According to the instruction, the relative quantification was applied to normalise the expression of the target genes to GAPDH using the Delta Delta Ct method. The primer sequences and the related parameters for the target genes are listed.

| Genes | Forward primer | Reverse primer |
| --- | --- | --- |
| *PTH1R* | CAATATCGTCCGGGTGCTCG | ACCAGCGTGGATTTGAGCAG |
| *RUNX2* | GATGACACTGCCACCTCTGAC | GGGATGAAATGCTTGGGAAC |
| *ALP* | GACCTCCTCGGAAGACACTC | TGAAGGGCTTCTTGTCTGTG |
| *Col 1α1* | GAGGGCCAAGACGAAGACATC | CAGATCACGTCATCGCACAAC |
| *OSX* | CCTCCTCAGCTCACCTTCTC | GTTGGGAGCCCAAATAGAAA |
| *OPN* | ATGATGGCCGAGGTGATAGT | ACCATTCAACTCCTCGCTTT |
| *GAPDH* | CGACAGTCAGCCGCATCTT | CCAATACGACCAAATCCGTTG |

**Western blotting (WB) analysis**

MG63 cells were harvested and lysed in RIPA buffer containing proteinase inhibitors. The protein samples were collected and separated by sodium dodecyl sulfate-polyacrylamide gel electrophoresis (SDS-PAGE) on 5% Tris–HCl reduced gels, then transferred to a PVDF membrane (Millipore). The membrane was blocked by incubating in a blocking solution containing 5% skim milk (BD) and 0.1% TWEEN 20 (Fluka Chemika) for 2 h. After that, the sheets were incubated with PTH1R (MA5-38295, Invitrogen), RUNX2 (ab23981, Abcam), ALP (ab229126, Abcam), OSX (ab209484, Abcam), OPN (ab283656, Abcam), p-PI3K (182651, Abcam), p-AKT (38449, Abcam), p-mTOR (5536, CST), PI3K (4257, CST), AKT (R23412, Zenbio), mTOR (R380411, Zenbio), GAPDH (ab181602, Abacm) and β-Actin (GB15003-100, Servicebio) at 4 °C overnight. Then, HRP-anti-Rb antibody (050884, KPL) was used for staining of PTH1R, RUNX2, ALP, OSX, OPN, p-PI3K, p-AKT, p-mTOR, PI3K, AKT, mTOR, GAPDH and β-Actin. Band densities on Western blots were assessed using Quantity One software (Versa Doc 5000, BioRad) and normalised to GAPDH proteins.

**Measurement of intracellular cAMP after treatment with PTH**

Transfected MG63 cells were treated for 15 min with 25 nmol/L PTH_1-34_ (GlpBio) or 50 µmol/L forskolin (MedChemExpress) in the presence of 1 mmol/L IBMX (3-isobutyl-1 methylxanthine, MedChemExpress). The amount of cAMP was determined with a cAMP Enzyme-linked Immunosorbent Assay (ELISA) kit (MEIMIAN).

**RNA sequencing and data analysis**

Total RNA was isolated from GFP, Wild and E302K stimulated with PTH with TRIzol reagent. RNA-seq libraries were prepared with the NEBNext® Ultra™ RNA Library Prep Kit (NEB, USA) and sequencing with Illumina Sequencing at Novogene Co. Ltd. (Beijing, China). Feature counts (v1.5.0-p3) were used to calculate read counts; the gene expression levels were calculated as the expected number of Fragments Per Kilobase of transcript sequence per Millions base pairs sequenced (FPKM) and read count mapped to this gene. The DESeq2 R package (1.20.0) was used to analyse differential expression. Genes with a corrected p-value ≤ 0.05 and an absolute log2 (fold-change) > 2 were considered differentially expressed. Gene Ontology (GO) enrichment analysis was performed for functional enrichment analysis. Kyoto Encyclopedia of Genes and Genomes (KEGG) enrichment analysis was performed for pathway enrichment analysis. Gene set enrichment analysis (GSEA) was used to analyse gene pathways/datasets. The corrected P-value≤0.05 was statistically significant.

**Statistical analysis**

Data are presented as the mean ± standard deviation (SD). Differences between the values were evaluated using a one-way analysis of variance (ANOVA; Tukey’s post-hoc test) or two-tailed Student’s t-test. Data are presented as means with a 95% confidence interval. P < 0.05 was considered statistically significant.

**
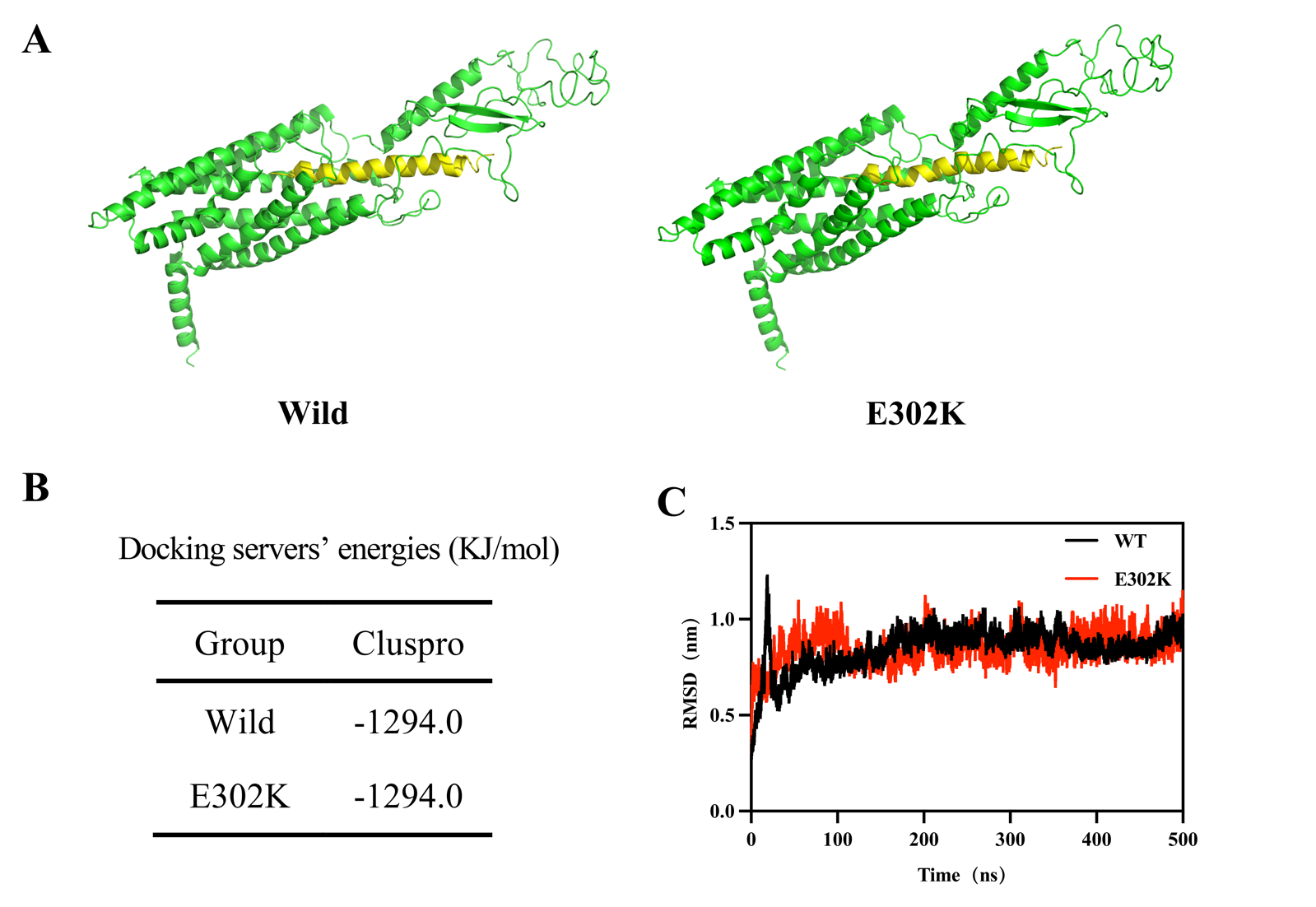
**

**Supplementary Figure 1.** The E302K mutation did not affect the binding affinity of PTH to the PTH1R protein. (A) Three-dimensional structure of PTH-PTH1R complexes. (B) Docking servers' energies (KJ/mol) for wild type and E302K variants. (C) Root-mean-square deviation (RMSD) curves during 500 ns MD simulations for PTH-PTH1R complexes.

**References**

1. Ioannidis NM, Rothstein JH, Pejaver V, et al. REVEL: An Ensemble Method for Predicting the Pathogenicity of Rare Missense Variants. Am J Hum Genet. 2016;99:877-885.

2. Schwarz JM, Cooper DN, Schuelke M, Seelow D. MutationTaster2: mutation prediction for the deep-sequencing age. Nat Methods. 2014;11:361-362.

3. Adzhubei IA, Schmidt S, Peshkin L, et al. A method and server for predicting damaging missense mutations. Nat Methods. 2010;7:248-249.

4. Ng PC, Henikoff S. Predicting deleterious amino acid substitutions. Genome Res. 2001;11:863-874.

5. Reva B, Antipin Y, Sander C. Predicting the functional impact of protein mutations: application to cancer genomics. Nucleic Acids Res. 2011;39:e118.

6. Choi Y, Chan AP. PROVEAN web server: a tool to predict the functional effect of amino acid substitutions and indels. Bioinformatics. 2015;31:2745-2747.

7. Liu X, Wu C, Li C, Boerwinkle E. dbNSFP v3.0: A One-Stop Database of Functional Predictions and Annotations for Human Nonsynonymous and Splice-Site SNVs. Hum Mutat. 2016;37:235-241.

8. Honorato RV, Koukos PI, Jimenez-Garcia B, et al. Structural Biology in the Clouds: The WeNMR-EOSC Ecosystem. Front Mol Biosci. 2021;8:729513.

9. Desta IT, Porter KA, Xia B, Kozakov D, Vajda S. Performance and Its Limits in Rigid Body Protein-Protein Docking. Structure. 2020;28:1071-1081 e1073.

10. Remmert M, Biegert A, Hauser A, Soding J. HHblits: lightning-fast iterative protein sequence searching by HMM-HMM alignment. Nat Methods. 2011;9:173-175.

11. Jimenez-Garcia B, Pons C, Fernandez-Recio J. pyDockWEB: a web server for rigid-body protein-protein docking using electrostatics and desolvation scoring. Bioinformatics. 2013;29:1698-1699.

12. Meng EC, Goddard TD, Pettersen EF, et al. UCSF ChimeraX: Tools for structure building and analysis. Protein Sci. 2023;32:e4792.

13. Waterhouse A, Bertoni M, Bienert S, et al. SWISS-MODEL: homology modelling of protein structures and complexes. Nucleic Acids Res. 2018;46:W296-W303.

14. Laskowski RA, Swindells MB. LigPlot+: multiple ligand-protein interaction diagrams for drug discovery. J Chem Inf Model. 2011;51:2778-2786.

15. Laskowski RA, Thornton JM. PDBsum extras: SARS-CoV-2 and AlphaFold models. Protein Sci. 2022;31:283-289.
